# Acute Care Surgery Models Worldwide: A Systematic Review

**DOI:** 10.1007/s00268-020-05536-9

**Published:** 2020-05-06

**Authors:** Mats J. L. van der Wee, Gwendolyn van der Wilden, Rigo Hoencamp

**Affiliations:** 1 Alrijne Hospital, Leiderdorp, The Netherlands; 2 Leiden University Medical Center, Leiden, The Netherlands; 3 Defense Healthcare Organization, Ministry of Defense, Utrecht, The Netherlands; 4 Erasmus University Medical Center, Rotterdam, The Netherlands

## Abstract

**Background:**

The Acute Care Surgery (ACS) model was developed as a dedicated service for the provision of 24/7 nontrauma emergency surgical care. This systematic review investigated which components are essential in an ACS model and the state of implementation of ACS models worldwide.

**Methods:**

A literature search was conducted using PubMed, MEDLINE, EMBASE, Cochrane library, and Web of Science databases. All relevant data of ACS models were extracted from included articles.

**Results:**

The search identified 62 articles describing ACS models in 13 countries. The majority consist of a dedicated nontrauma emergency surgical service, with daytime on-site attending coverage (cleared from elective duties), and 24/7 in-house resident coverage. Emergency department coverage and operating room access varied widely. Critical care is fully embedded in the original US model as part of the acute care chain (ACC), but is still a separate unit in most other countries. While in most European countries, ACS is not a recognized specialty yet, there is a tendency toward more structured acute care.

**Conclusions:**

Large national and international heterogeneity exists in the structure and components of the ACS model. Critical care is still a separate component in most systems, although it is an essential part of the ACC to provide the best pre-, intra- and postoperative care of the physiologically deranged patient. Universal acceptance of one global ACS model seems challenging; however, a global consensus on essential components would benefit any healthcare system.

## Introduction

Delivering adequate healthcare to the acutely ill surgical patient has been a challenge for decades. Over the years, the quality of acute care improved significantly. However, due to increasing numbers of patients presenting to the emergency department (ED), analysis and distribution of resources has become even more important [1, 2]. In response to the lack of dedicated and well-organized services for the provision of non-traumatic emergency surgical care, the American Association for the Surgery of Trauma (AAST) initiated the development of the Acute Care Surgery (ACS) model, which was subsequently adopted in most institutions offering emergency surgical care across the United States (US) [3].

Initially, most high-income countries worldwide had a traditional on-call model, comprising of a rotating pool of surgeons managing most or all emergency surgical caseload in addition to elective duties [4]. No dedicated team was available, the surgeon on-call was often not on-site, and most emergency surgery was performed either in after-hours when an operating room (OR) was available, or elective cases were canceled in order to perform those interventions.

This changed with the implementation of the original (US) ACS model, with fundamental components like a dedicated surgical team (surgeon, residents, nursing staff) separated from other surgical services, and the inclusion of surgical critical care. Resources, infrastructure, and surgical skills were combined to provide care for all surgical emergencies 24/7 [5–8]. Hence, the attending surgeon staffing the ACS service today is accountable for the whole Acute Care Chain (ACC), being broadly trained in emergency general surgery, trauma surgery, and critical care. Thus, concerns regarding the increasing subspecialization of surgeons, and subsequent decline in expertise and quality of care for general surgical emergencies are attacked [3]. Furthermore, the ACS model counteracted the decreased interest in trauma surgery due to the increasing non-operative nature of the field, by integrating trauma with emergency general surgery, thereby increasing the trauma surgeon’s operative workload and clinical productivity [5, 8–13].

The model has shown to be a necessary addition to the healthcare system with improved patient outcomes and cost-effectiveness [4, 6, 7, 13–20]. Several variations of this original ACS model have gained popularity around the world [21]. However, the structure of the different models varies broadly and it remains unclear which components constitute an optimal model, and whether this model could be uniformly implemented worldwide. The aim of this systematic review is to investigate which components are essential for a uniform ACS model, by giving an overview of the current available ACS models worldwide and their state of implementation.

## Materials and methods

This systematic literature review was performed using the guidelines of the Preferred Reporting Items for Systematic Reviews and Meta-Analyses statement (PRISMA) [22]. Methods, inclusion criteria, and objectives were gathered in a protocol and registered in PROSPERO (ID: CRD42019118449).

### Search strategy

A literature search was conducted using PubMed, MEDLINE, EMBASE, Cochrane library, and Web of Science databases. An additional literature search was conducted to identify relevant meeting abstracts. The search strategy was devised with the help of a medical librarian expert from Leiden University Medical Center. The final search was performed on 11 September 2018. The search terms included ''acute care surgery,’’ ''acs,’’ ''emergency surgery,’’ ''es,’’ ''worldwide,’’ ''systems,’’ ''trauma and acute care,’’ ''economics.’’

### Selection of articles

Articles from January 2000 until September 2018 were included. Titles of articles identified by the search were screened for relevancy. Titles and abstracts of identified articles were then screened for relevancy. Any disagreement about the relevancy of titles and abstracts was resolved by discussion between the two reviewers (MVDW and GVDW), if needed with involvement of a third author (RH). The full text of included abstracts was retrieved. We included articles providing an extensive description of an ACS model, such as studies reporting on patient outcomes, surgeon satisfaction and opinion on ACS, cultural differences, and financial implications of ACS models. In addition, only articles in English and Dutch were included. Articles that exclusively focused on outcomes in pediatric or geriatric patients, education or training were excluded. Additionally, the reference lists of included articles were screened for relevant studies. We also included grey literature from websites of surgical societies, manuscripts, meeting abstracts, and additional literature received through contact with local experts. The search strategy for meeting abstracts is provided in Appendix 1

### Data extraction

Data extraction was performed by breaking down all models in relevant structural components, in a table using Microsoft® Excel version 16.23.

**Table:** 

Relevant structural components of ACS models
∙ Region/country
∙ Type of model
∙ Dedicated team: yes/no
∙ Dedicated unit: yes/no
∙ Elective duties of attending surgeon
∙ Dedicated operating room (OR) access
∙ Service coverage
∙ ED coverage
∙ Trauma coverage
∙ Critical care coverage

### Quality assessment

No quality-assessment tool for descriptive literature exists to our knowledge. The Newcastle–Ottawa Scale (NOS) is a validated tool designed for assessing the quality of nonrandomized studies, but not specifically descriptive research [23]. We found the NOS the most suitable tool to assess quality of included studies. Two authors (MVDW and GVDW) independently assessed study quality. Any discrepancies were resolved by consensus discussion, with involvement of a third author (RH) if needed. Study quality was rated ''high,'' ''medium’’ or ''low’’ according to points awarded for each domain [24]. The complete NOS scores are provided in Appendix 2.

## Results

### Study characteristics

**Fig. 1:**
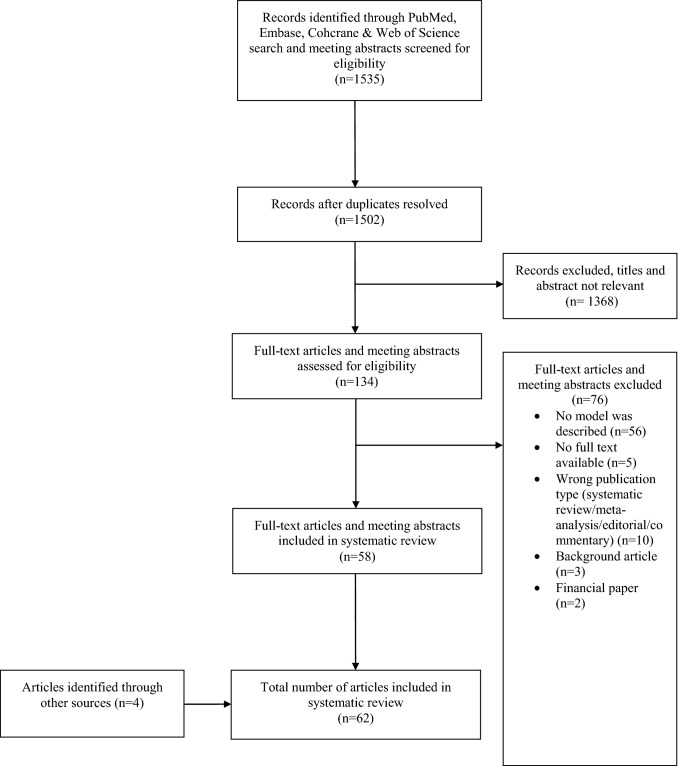
Flowchart of included studies

**Fig. 2:**
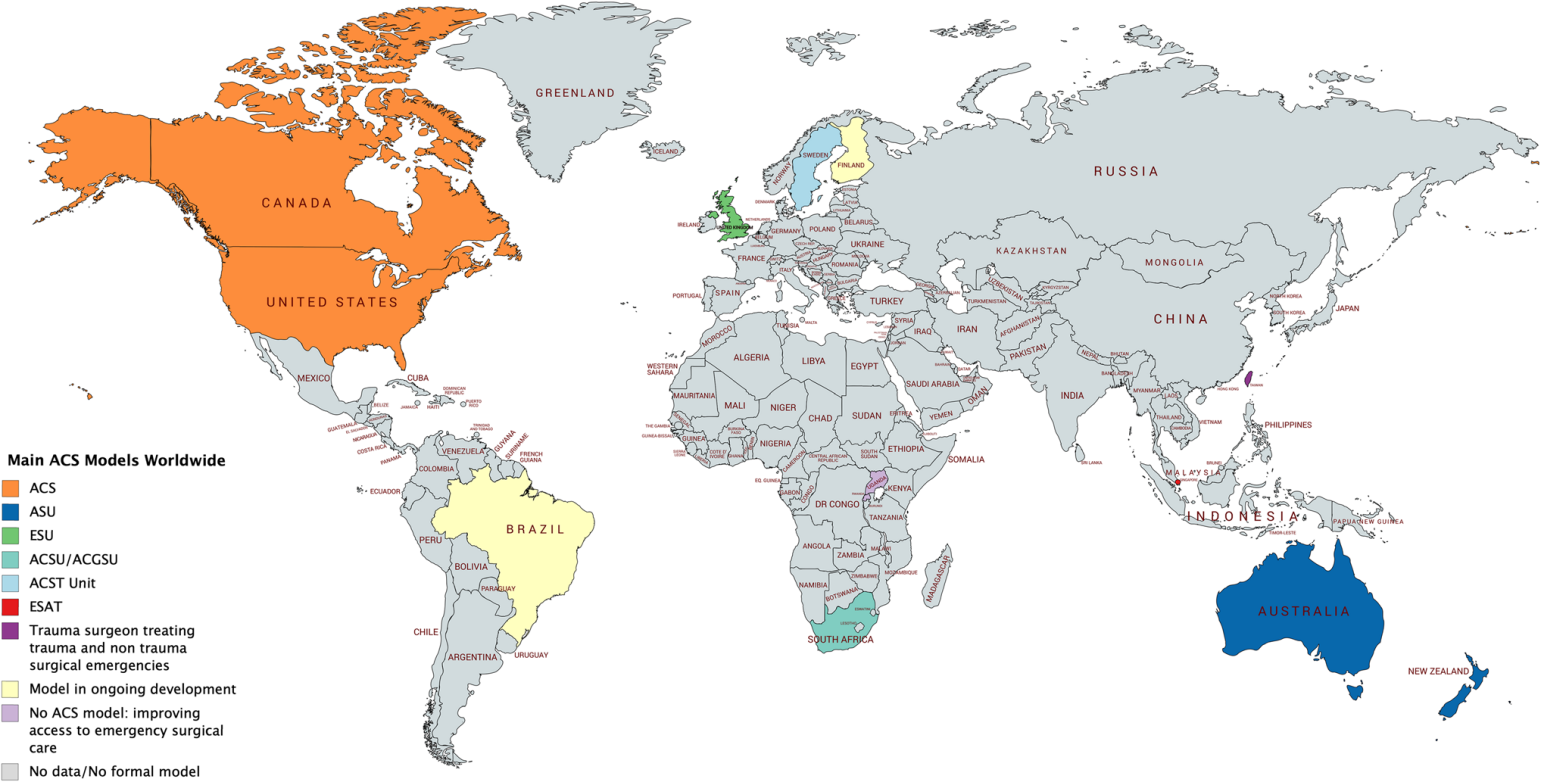
Main ACS models worldwide

The search identified 1292 articles; another 243 meeting abstracts were identified through an additional search. After removal of duplicates, 1502 abstracts were screened, and 134 full-text articles were evaluated after removal of irrelevant abstracts. After applying exclusion criteria, 58 full-text articles and meeting abstracts were eligible for inclusion, as well as four articles from additional sources (grey literature). In total, 62 articles describing ACS model-variations in 13 countries were included (Figs. 1, 2 and Tables 1, 2). The structural components of the model described in each article are summarized in Table 2.

**Table 1:** Demographics of included studies

Author	Year	Country	Study design	Model	NOS Score	Quality rating
Beardsley et al. [37]	2013	Australia	Retrospective	SAPU	6	Low
Cox et al. [38]	2010	Australia	Report	ASU	–	–
Dickfos et al. [39]	2017	Australia	Retrospective	RAMS	5	Low
Lancashire [43]	2014	Australia	Retrospective	ASU	6	Low
Allaway et al. [36]	2017	Australia	Retrospective	ASU	6	Low
Gandy et al. [40]	2010	Australia	Retrospective	ACS	6	Low
Guy et al. [41]	2018	Australia	Retrospective	ASU	6	Low
Kinnear et al. [42]	2017	Australia	Retrospective	ASU	7	Med
Lehane et al. [44]	2010	Australia	Retrospective	ACS	6	Med
Musiienko et al. [45]	2016	Australia	Retrospective	ASU	8	High
Parasyn et al. [46]	2009	Australia	Retrospective	ACS	5	Low
Pepingco et al. [47]	2012	Australia	Retrospective	ASU	6	Low
Shakerian et al. (Br J Surg) [49]	2015	Australia	Retrospective	ASU	8	High
Shakerian et al. (2) (World J Surg) [48]	2015	Australia	Retrospective	ASU	8	High
Suhardja et al. [50]	2015	Australia	Retrospective	ASU	6	Low
Von Conrady et al. [51]	2010	Australia	Retrospective	ASU	6	Low
Wang et al. [52]	2018	Australia	Financial analysis	ASU	–	–
Suen et al. [53]	2013	Australia	Retrospective	EGS	6	Low
Poggetti et al. [35]	2009	Bra/Fin/USA	Descriptive	–	–	–
Anantha et al. [29]	2015	Canada	Retrospective	ACCESS	6	Low
DeGirolamo et al. [30]	2018	Canada	Multicenter observational	EGS	–	–
Faryniuk et al. [31]	2013	Canada	Retrospective	ACSS	6	Low
Kreindler et al. [32]	2012	Canada	Retrospective	ACS	7	Med
Lim et al. [9]	2013	Canada	Retrospective	ACS	6	Low
Qureshi et al. [15]	2013	Canada	Pre–post	ACCESS	6	Low
Van Zyl et al. [33]	2018	Canada	Prospective	ACS	8	High
Wanis et al. [34]	2014	Canada	Retrospective	ACS	6	Low
Hsee et al. (World J Surg) [54]	2012	New Zealand	Retrospective	ASU	6	low
Hsee et al. (ANZ J Surg) [55]	2012	New Zealand	Descriptive	ASU	–	–
Pillai et al. [56]	2013	New Zealand	Retrospective	ASU	6	Low
Poh et al. [57]	2013	New Zealand	Retrospective	ASU	6	Low
Poole et al. [58]	2011	New Zealand	Descriptive	ACS team	–	–
Mpirimbanyi et al. [69]	2017	Rwanda	Cross-sectional	–	–	–
Mathur et al. [65]	2018	Singapore	Retrospective	ESAT	6	Low
Al Ayoubi et al. [64]	2012	Sweden	Quality control	ACST Unit	–	–
Fu et al. [66]	2014	Taiwan	Pre–post	ACS	6	Low
Dresser et al. [70]	2017	Uganda	Descriptive	ECP	6	Low
Bokhari et al. [59]	2015	UK	Audit	ESU	6	Low
Bokhari et al. [60]	2016	UK	Retrospective	ESU	7	Med
Navarro et al. [61]	2017	UK	Retrospective	STU	6	Low
Sorelli et al. [62]	2008	UK	Retrospective	Dedicated EGS surgeon	6	Low
Tincknell et al. [63]	2009	UK	Audit	EST	–	–
Santry et al. [26]	2015	USA	Survey	ACS/On-call/Hybrid	–	–
Austin et al. [5]	2005	USA	Retrospective	EGS	6	Low
Barnes et al. [10]	2011	USA	Retrospective and questionnaire	ACS	–	–
Britt et al. [6]	2009	USA	Retrospective	ACS	6	Low
Bruns et al. [13]	2016	USA	Retrospective	ACES	5	Low
Cherry-Bukowiec et al. [12]	2012	USA	Retrospective	NTE	6	Low
Ciesla et al. [7]	2011	USA	Retrospective	ACS	–	–
Cubas et al. [14]	2012	USA	Retrospective	ACS	6	Low
Diaz et al. [16]	2011	USA	Retrospective	ACS	6	Low
Ekeh et al. [17]	2008	USA	Retrospective	ACS	6	Low
Garland et al. [27]	2007	USA	Retrospective	ACS	–	–
Ladhani et al. [28]	2018	USA	Retrospective	EGS	7	Med
Matsushima et al. [8]	2011	USA	Retrospective	ACS	8	High
Miller et al. [18]	2012	USA	Retrospective	ACS	4	Low
Procter et al. [19]	2013	USA	Financial analysis	ACS	–	–
Pryor et al. [20]	2004	USA	Retrospective	EGS	6	Low
Santry et al. [25]	2014	USA	Interview analysis	ACS	–	–
Sweeting et al. [11]	2013	USA	Financial analysis	ACS	–	–
Moodie [68]	2015	RSA	Audit	ACGSU	–	–
Klopper et al. [67]	2017	RSA	Retrospective	ACGSU	–	–

NOS, Newcastle–Ottawa Scale (study designs other than case–control –or cohort studies could not be scored using the Newcastle–Ottawa Scale); SAPU, Surgical Assessment and Planning Unit; ASU, Acute Surgical Unit; ACS, Acute Care Surgery; RAMS, Rapid Assessment Medical Surgical Unit; EGS, emergency general surgery service; ACCESS, Acute Care Emergency Surgery Service; ACSS, acute care surgical service; ESAT, Emergency Surgery and Trauma Team; ACST, Acute Care Surgery and Trauma; ECP, emergency care practitioner; ESU, emergency surgical unit; STU, Surgical Triage Unit; EST, emergency surgical team; ACES, NTE, Nontrauma Emergency Surgery service; ACGSU, acute care and general surgical unit; ANZ J Surg, ANZ Journal of Surgery; World J Surg, World Journal of Surgery; Retrospective, Retrospective cohort study

**Table 2:** Components of ACS models worldwide

Region/country	ACS model	Dedicated team	Dedicated unit	Elective duties surgeon	Dedicated OR access	Coverage				ED coverage	Trauma coverage	Critical care coverage
						Daytime		Night				
						Sur	Res	Sur	Res			
*North America*												
USA	ACS [5–8, 10, 11, 13, 14, 16–19, 25–28]	Yes	Varied	Varied	Varied	In-house	Not reported*	In-house	Not reported*	Varied	Included	Included
	NTE [12]	Yes	Not reported*	Not reported*	Not reported*	In-house	Not reported*	Not reported*	Not reported*	Not reported*	Not included	Not included
	EGS and trauma service [20]	Yes	Yes	Not reported*	Not reported*	In-house	In-house	On-call	In-house	Yes	Included	Included
Canada	ACS/ACCESS [9, 15, 29–34]	Yes	No	Daytime	Daytime	In-house *	Not reported*	Not reported*	Not reported*	No	Not included	Not reported*
*South America*												
Brazil [35]	None	No	No	Not reported*	Not reported*	Not reported*	Not reported*	Not reported*	Not reported*	Not reported*	Included	Not included
*Australasia*												
Australia/New Zealand	ASU (consultant led) [36, 38, 41–43, 45–52]	Yes	No	Cleared	Yes	In-house	In-house	On-call	In-house	Not reported*	Varied	Not included
Australia	SAPU [37]	Yes	Yes	Not reported*	Yes	On- call	In-house	On-call	In-house	Yes	Not reported*	Not reported*
Australia	RAMS [39]	No	Yes	No	No	On-call	Not reported*	Not reported*	Not reported*	Not reported*	Not included	Not Reported*
Australia	ACS/EGS service (consultant led) [40, 44, 46, 53]	Yes	Yes	Yes	Yes	In-house	In-house/not reported*	No/on-call	On-call/not reported*	Yes/Not reported*	Not included/not reported*	Not reported*
*Europe*												
United Kingdom	ESU [59, 60]	Yes	Yes	Yes	Yes	In-house	Not reported*	On-call	Not reported*	Not reported*	Not included	Not reported*
	STU [61]	Yes	Yes	n/a**	n/a**	In-house	In-house	On-call	In-house	Yes	n/a**	n/a**
	Single dedicated EGS surgeon [62]	Yes	Yes	Yes	Yes	In-house	In-house	On-call	Not reported*	Yes	Not reported*	Not reported*
	EST [63]	Yes	Yes	Yes	Yes	In-house	In-house	On-call	In-house	On-call	Not included	Not reported*
Sweden	ACST Unit [64]	Yes	Yes	Shared	Shared	In-house	In-house	On-call	In-house	Yes	Included	Included
Finland	Traditional on-call [35]	–	–	–	–	–	–	–	–	–	–	–
*Asia*												
Singapore	ESAT [65]	Yes	Yes	Not reported*	Not reported*	In-house	Not reported*	On-call	Not reported*	Not reported*	Included	Not reported*
Taiwan	ACS (single surgeon) [66]	No	No	Not reported*	Not reported*	In-house	Not reported*	In-house	Not reported*	Yes	Included	Not reported*
*Africa*												
South Africa	ACGSU [67, 68]	Yes	Yes	No	No	In-house	In-house	On-call	In-house	Yes	Not included	Not included
Rwanda	None [69]	–	–	–	–	–	–	–	–	–	–	–
Uganda	None (ECP) [70]	–	–	–	–	–	–	–	–	–	–	–

ACS, Acute Care Surgery; Sur, attending surgeon; Res, resident; ED, emergency department; OR, operating room; NTE, nontrauma emergency service; ASU, Acute Surgical Unit; SAPU, Surgical Assessment and Planning Unit; RAMS, Rapid Assessment Medical Surgical Unit; EGS, emergency general surgery (service); ACCESS, Acute Care Emergency Surgery Service; ESU, emergency surgical unit; STU, Surgical Triage Unit; EST, emergency surgical team; ACST, Acute Care Surgery and Trauma; ECP, emergency care practitioner; ESAT, Emergency Surgery and Trauma Team; ACGSU, acute care and general surgical unit

In-house: surgeon/resident is on-call on site

On-call: surgeon/resident is on-call but not on site

Dedicated team: Separate surgical team with attending service director, attending surgeons, residents and assistants, dedicated to the provision of ACS

Dedicated unit: ACS team has a separate (sub)unit or ward. ED coverage: emergency surgery team is concerned with the initial assessment or surgical consultation of patients in the Emergency Department

*Not reported: it is unknown whether a structural feature is part of a model because it is was not reported on in included articles; No: structural feature was described in included articles but not part of the model

**STU is a triage unit and does not perform interventions

### North America

Eighteen studies described ACS models in the USA [5–8, 10–14, 16–20, 25–28]. The majority of studies described a dedicated ACS service with daytime on-site attending coverage, and dedicated resident rotations [5, 7, 8, 10, 11, 14, 28]. Most models provided trauma [7, 8, 10, 11, 14, 17–20, 25, 27]—and/or critical care [6, 7, 10, 11, 18–20, 25, 27], seven studies reported a completely separate service or subunit [5–7, 10, 16, 19, 20]. The elective duties of attending surgeons were cleared in seven, [5, 6, 12–14, 20, 28] eight had protected operating room (OR) time, [6, 8, 11, 13, 14, 19, 26, 27], and six provided ED coverage by attendings and/or residents [5–7, 14, 17, 20]. These components were not frequently described in other articles. Only two articles reported ACS surgeons were trained to provide critical care but did not specifically describe ICU coverage [26, 28].

Eight studies discussed ACS models in Canada [9, 15, 29–34]. The majority of the articles described a dedicated ACS service with on-site daytime attending coverage in which the attending surgeon was cleared of elective duties, exclusively providing non-traumatic emergency surgical care and daytime protected OR time, varying from 5 to 8 h per day. Other structural features of ACS models reported in these articles included a service that solely consisted of a dedicated surgeon [29, 31, 34], on-site night-time attending coverage [9, 33], 24-hour resident coverage [9]. Two articles described a separate (sub)unit for the ACS service. In four articles, the ACS team was responsible for ED emergency surgical consultations [15, 29, 31, 33]. Critical care was not described as an ACS component in any of the included articles.

### South America

Poggetti et al. [35] reported on the early development of an ACS model in Brazil. No dedicated ACS model was described, only specialists working in-house 12 to 24-hour shifts, covering trauma and nontrauma emergency surgical services. Critical care is provided separately by anesthetists or specialists trained in critical care.

### Australasia

Twenty-three articles from Australasia (Australia and New Zealand) described Acute Surgical Unit (ASU) models for the provision of acute care surgery [36–58]. ASU features that were repeatedly mentioned included a dedicated, consultant (attending)-led ACS service, with clearance of the attending surgeon’s elective workload, daytime on-site attending coverage, 24/7 coverage by dedicated residents, and on-call from home night-time attending coverage. All New Zealand articles reported 24/7 dedicated OR access, whereas Australian articles mainly reported daytime or shared protected OR time [37, 38, 40–46, 50–53]. None of the included articles reported on-site night-time attending coverage of an ASU. Six of the ASU’s described were a separate (sub)unit from other surgical services [36–38, 41–43]. Six articles described coverage of the ED by the ASU team or resident during working hours [37, 38, 43, 46, 48, 49]. None of the articles reported ICU coverage or provision of critical care. Trauma care was reported in 4 articles [38, 47–49].

### Europe

#### United Kingdom (UK)

Five articles described ACS models in the UK [59–63]. Two articles described the same Emergency Surgical Unit (ESU) model [59, 60]. The majority of the articles described a dedicated team operating within an independent (sub)unit, with daytime on-site attending coverage provided by a surgeon without elective duties, night-time on-call attending coverage, and round-the-clock coverage by dedicated residents. Four articles reported dedicated OR access, predominantly via a shared or attending-controlled OR list [59, 60, 62, 63]. One article reported attending coverage of the ED [61], but another article described a Surgical Assessment Unit (SAU) where patients are assessed by the attending [62]. None of the articles reported critical care or trauma care to be provided by the ACS service. One article described a surgical triage unit (STU) aimed at improving clinical efficiency by assessing and triaging surgical patients [61].

#### Continental Europe

Two articles reported on ACS models in Scandinavia [35, 64]. One article from Sweden described a dedicated ACS unit separated from other services with a 28-bed acute surgical ward, with attendings cleared from elective workload, daytime on-site attending coverage, 24/7 on-site coverage by residents dedicated to the unit, night-time on-call attending coverage, and shared dedicated OR time. Furthermore, the unit provided ED, ICU, and trauma coverage. The article from Finland did not describe an existing ACS model. Emergency surgical care is provided by all university—and central hospitals, via a traditional on-call model or by 24 h in-house specialists from large surgical specialties. These surgeons do not provide critical care.

### Asia

Two articles were found, from Singapore and Taiwan, respectively [65, 66]. The current model in Singapore consists of a consultant (attending)-led, dedicated emergency surgery and trauma team (ESAT), with an in-house attending cleared from elective duties and present during daytime. This model includes a separate ward and trauma coverage. Resident coverage, OR access, ED, and critical care coverage were not described. In Taiwan, a 24/7 in-house trauma surgeon, who is not cleared from clinical duties covering all trauma and non-trauma surgical emergencies while also covering the ED, was described. No separate ward, OR access, nor critical care was described.

### Africa

Two studies described an acute care and general surgical unit (ACGSU) at the same hospital in South Africa [67, 68]. It consists of a dedicated, separate unit with an independent ward, and round-the-clock resident coverage by dedicated residents who are supported by on-call attendings. No dedicated OR time is available. The unit covers the ED, but does not provide critical care or trauma care.

No comprehensive ACS model was in place in Rwanda and Uganda [69, 70].

## Discussion

Our systematic review provides a comprehensive overview outlining the structural features of the different ACS models implemented worldwide, thereby determining which components are essential to comprise one uniform system and whether that would be desirable.

Worldwide, a transition in the acute care chain is seen, with adoption of various ACS models in high-income countries for the structured and dedicated provision of emergency general surgical care. However, we found that extensive national and international heterogeneity exists in the structure of ACS models, most likely due to discrepancies in healthcare environment, hospital infrastructure, and available resources [26]. We identified relevant structural components of ACS services using the criteria for ACS models formulated by the AAST Committee for Acute Care Surgery, the GSA 12-point plan (Table 3), and components frequently reported in the ACS literature (Table 2) [3, 71].

**Table 3:** General Surgeons Australia 12-point plan for Emergency General Surgery [71]

1	Emergency general surgery is a continuing core competency of a general surgeon
2	Emergency general surgery should be consultant led
3	There should be dedicated staff allocated to the provision of emergency care, with the need for training recognized
4	There should be separation of emergency general surgery and elective general surgery systems
5	There should be appropriate and timely access to emergency operating theaters
6	Emergency operations should be performed during the working day unless there is threat to life, limb, or organ
7	Consultant (attending) surgeons should contribute to the efficient management of emergency theater
8	The period of service of the emergency general surgeon must be defined. Work practices must reflect safe hours principles
9	There must be robust handover and transfer of care: peer to peer, documented and retrievable
10	Best practice should be defined. Quality should be measured by clinically meaningful Key Performance Indicators (KPI’s)
11	The service must reflect community need and regional variation
12	The service must be valued (recognized, rewarded, resourced, and renumerated)

Previous systematic reviews have focused on clinical and financial outcomes of ACS models [21]. A recent systematic review from New Zealand compared ACS models in Australasia, UK, and Europe using the General Surgeons Australia’s (GSA) 12-point plan (Table 3), but only included a few hospitals and their specific models [72].

Components included in a majority of the models were a dedicated surgical service covering all non-trauma emergency surgery, with daytime on-site attending coverage, clearance of attending’s elective duties, and 24/7 coverage by dedicated residents. (Table 2) Round-the-clock on-site attending coverage, one of the initial aims of the ACS model designed by the AAST, was only reported in articles from the USA and the article from Taiwan [3]. ACS wards or (sub)units separated from other surgical services were reported in the UK, Sweden, South Africa, and Singapore.

Trauma care was only frequently reported in articles from the USA. In Canada, ACS services exclusively cover non-traumatic surgical emergencies [4]. This is in contrast with the model in the USA, which revolves around an acute and critical care trained trauma surgeon, and hence, logically, covers trauma. However, in Canada, ACS is mostly provided by general surgeons. The latter is also the case in Australasia, the UK, South Africa, Singapore, and Sweden.

Except for South Africa, emergency surgery models are not implemented yet in Africa; their focus is overall access to (emergency) healthcare, by improving infrastructure and availability of resources.

Critical care was added as an important entity within the original ACS model; completing the acute care chain (ACC). Although important in the US models, it is structurally missing or not reported in articles from other countries, including Canada [3]. In our vision, it is essential to the concept of ACS that a patient is being followed from arrival in the ED up until discharge, covering the full spectrum of care for acutely ill surgical patients. Peri-operatively, these acutely ill patients are in a state of survival. Peri-operative management of these patients focuses on damage control and powerful resuscitation. Therefore, critical care is a necessary component of the ACC, providing the full range of treatment for these physiologically deranged surgical patients. Hence, ACS surgeons should also be trained in that part of the pathophysiology.

OR access was only regularly described in Australasia, UK, and Sweden. In addition, if reported, it varied from shared access or a few hours per day, to 24/7 access (only in New Zealand). In the USA, only eight articles mentioned protected OR time, although it is a standard component of the original ACS model. ED coverage was reported in Sweden, South Africa, and Taiwan. In our opinion, both dedicated OR access and ED coverage are a key component to streamline clinical care delivery and improve quality of care. Similar to the critical care component, these components are essential to complete the ACC. Such a structure would ensure rapid assessment and management of acute surgical patients, decreased after-hours operating, and thus improved quality of care.

Although the rationale for the development of an ACS model also exists in Europe, healthcare systems in Europe are still lacking a dedicated model. Uranues performed a survey including 18 countries, to determine whether a European ACS model exists [73]. They reported that it did not, and that ACS is not recognized as a separate specialty. Models involving emergency surgery are developed in line with country-specific factors, such as the political and socioeconomic situation and varied extensively within countries. In addition, the article reported varying levels of support for the model in participating countries. In the majority of the European countries, surgical emergencies are managed by surgical subspecialists according to the type of emergency (e.g., abdominal, trauma, etc.). No distinction was made between trauma and non-trauma in the management of surgical emergencies. Furthermore, elective and emergency surgical work streams are not separated in most European centers, and there are no dedicated resources for acute care surgery [73]. Hence, there is no consensus on whether an ACS system and ACS as a subspecialty are desirable, and if so, in what form. One of the reasons might be the difference in the specialty of trauma surgery. In continental Europe, trauma surgery comprises both skeletal and visceral trauma, whereas in other countries, including the USA, it only includes visceral trauma (skeletal trauma is part of the orthopedics department). That difference results in the question which surgeon should take the role of acute care surgeon. It is debatable whether ACS should be part of the gastro-intestinal department instead of the trauma department [73]. All difficulties aside, there is some movement toward a structured ACS model in Spain and Scandinavia according to reports there [64, 74].

A possibility for an optimal, unified European model may be in line with the GSA 12-point plan, in which general surgeons provide emergency surgery, meaning that both GI- and trauma surgeons could participate in the model with additional training in managing the acutely ill surgical patient. In our vision, a European ACS model should have the following fundamental components in order to provide a decent ACC: a dedicated surgical team managing all non-traumatic surgical emergencies, with 24/7 on-site attending (free from elective duties) -and resident coverage, round-the-clock access to a dedicated emergency operating room, and coverage of the ED and ICU by the ACS service. Most of these structural features have already been implemented in the Swedish ACST unit, which could serve as an example [64].

To assess whether an ACS model with the structure described above would be desirable, and (financially) viable in continental Europe, such a model should be piloted and evaluated first, before expanding nationwide. Our research group is currently performing a survey evaluating the state of implementation of ACS models in hospitals in the Netherlands.

## Limitations

Our review has several limitations. First of all, most included studies are of retrospective nature, and therefore at risk of selection and information bias. No ideal tool is available to perform quality assessment of the descriptive literature. The NOS was found to be most suitable, but it is difficult to draw conclusions about study quality based on this assessment. The majority of the studies were of low quality according to the NOS. However, our review focuses on the description of the ACS model, so the quality of the conducted research is less relevant. Furthermore, we may have missed relevant articles due to our language criterion. In addition, since the start of this review, new articles may have been published or existing models discussed in this review may have further developed. However, this systematic review is the only one of its scale identifying essential structural features of ACS models across all continents.

## Conclusion

In conclusion, ACS has variably been implemented in mostly high-income countries, and large national and international heterogeneity still exists in the structure and components of the model. Critical care is still a separate unit and specialty in most systems while it is essential to be part of the ACC in order to provide the best pre-, intra-, and postoperative care of the physiologically deranged patient. Universal acceptance of one global ACS model seems challenging; however, a global consensus on essential components (see the ACC components described above) would benefit any healthcare system that is considering implementing such a model.

## Appendix 1: Search strategy meeting abstracts (grey literature)

### Embase

http://ovidsp.ovid.com/ovidweb.cgi?T=JS&PAGE=main&MODE=ovid&D=oemezd

ACS as main subject, coupled to organization and administration-terms:

((“acute care surgery”.ti OR “acute care surgical”.ti OR “acute care surgeons”.ti OR “acute care surgeon”.ti OR “acs surgery”.ti OR “acs surgeons”.ti OR *”acute care surgery”/OR “emergency surgery”.ti OR “emergency surgical”.ti OR “emergency surgeon”.ti OR “emergency surgeons”.ti OR “emergency surgeries”.ti OR “emergency general surgery”.ti OR “emergency general surgeon”.ti OR “emergency general surgeons”.ti OR “acute trauma surgery”.ti OR “acute surgery”.ti OR “acute surgical”.ti OR “acute surgical care”.ti OR “acute surgical emergencies”.ti OR “acute surgical emergency”.ti OR “acute surgical admission”.ti OR “acute surgical admissions”.ti OR “acute surgical beds”.ti OR “acute surgical care”.ti OR “acute surgical emergencies”.ti OR “acute surgical emergency”.ti OR “acute surgical intervention”.ti OR “acute surgical interventions”.ti OR “acute surgical management”.ti OR “acute surgical model”.ti OR “acute surgical patient”.ti OR “acute surgical patients”.ti OR “acute surgical procedure”.ti OR “acute surgical procedures”.ti OR “acute surgical service”.ti OR “acute surgical services”.ti OR “acute surgical setting”.ti OR “acute surgical settings”.ti OR “acute surgical site”.ti OR “acute surgical specialties”.ti OR “acute surgical treatment”.ti OR “acute surgical unit”.ti OR “acute surgical units”.ti OR “acute surgical ward”.ti OR “acute surgical wards”.ti OR “surgical emergency”.ti OR “surgical emergencies”.ti OR “surgery emergencies”.ti OR “surgery emergency”.ti OR ((*”Emergency Treatment”/OR *”emergency care”/OR *”evidence based emergency medicine”/OR exp *”Emergency Health Service”/) AND (“Surgery Department”.ti OR *”General Surgery”/))) AND (exp “economics”/OR **exp** “**organization and administration”/**OR exp “standard”/OR “trend study”/OR “manpower”/OR “Theoretical Model”/OR “Educational Model”/OR “nonbiological model”/OR exp “Health Care Quality”/OR “Cost–Benefit Analysis”/OR “Physicians’ Practice Pattern*”.mp OR “Physicians Practice Pattern*”.mp OR “Physician Practice Pattern*”.mp OR “Outcome Assessment”/OR “Length of Stay”/OR “Hospital Readmission”/OR “Health Services Accessibility”.mp OR “Health Service Accessibility”.mp OR “Health Care Accessibility”.mp OR “Health Services Need*”.mp OR “Health Service Demand*”.mp OR “Health Service Need*”.mp OR “Health Services Demand*”.mp OR “Health Care Need*”.mp OR “Health Care Demand*”.mp OR “Clinical Competence”/OR “burden of disease”.mp OR exp “Disease Burden”/OR “model”.mp OR “models”.mp OR “resources”.mp OR “resource”.mp OR “implementation”.mp OR implement*.mp OR “competent”.mp OR “productivity”.mp OR “case mix”.mp OR “overcrowding”.mp OR overcrowd*.mp OR “timing”.mp OR “Time Factor”/OR “cost”.mp OR “costs”.mp OR “workforce”.mp OR “workforces”.mp OR “trauma systems”.mp OR “trauma system”.mp) AND exp “Humans”/AND (english.la OR dutch.la OR german.la) NOT ((“case report”/OR “case report”.ti) NOT (exp “Review”/OR “review”.ti))) AND (conference abstract).pt

### Web of Science

http://isiknowledge.com/wos

Advanced Search

ACS as main subject, coupled to organization and administration-terms:

ti = (“acute care surgery” OR “acute care surgical” OR “acute care surgeons” OR “acute care surgeon” OR “acs surgery” OR “acs surgeons” OR *”acute care surgery” OR “emergency surgery” OR “emergency surgical” OR “emergency surgeon” OR “emergency surgeons” OR “emergency surgeries” OR “emergency general surgery” OR “emergency general surgeon” OR “emergency general surgeons” OR “acute trauma surgery” OR “acute surgery” OR “acute surgical” OR “acute surgical care” OR “acute surgical emergencies” OR “acute surgical emergency” OR “acute surgical admission” OR “acute surgical admissions” OR “acute surgical beds” OR “acute surgical care” OR “acute surgical emergencies” OR “acute surgical emergency” OR “acute surgical intervention” OR “acute surgical interventions” OR “acute surgical management” OR “acute surgical model” OR “acute surgical patient” OR “acute surgical patients” OR “acute surgical procedure” OR “acute surgical procedures” OR “acute surgical service” OR “acute surgical services” OR “acute surgical setting” OR “acute surgical settings” OR “acute surgical site” OR “acute surgical specialties” OR “acute surgical treatment” OR “acute surgical unit” OR “acute surgical units” OR “acute surgical ward” OR “acute surgical wards” OR “surgical emergency” OR “surgical emergencies” OR “surgery emergencies” OR “surgery emergency” OR ((*”Emergency Treatment” OR *”emergency care” OR *”evidence based emergency medicine” OR “Emergency Health Service”) AND (“Surgery Department” OR *”General Surgery”))) AND ts = (“economics” OR “**organization and administration”** OR “standard” OR “trend study” OR “manpower” OR “Theoretical Model” OR “Educational Model” OR “nonbiological model” OR “Health Care Quality” OR “Cost–Benefit Analysis” OR “Physicians’ Practice Pattern*” OR “Physicians Practice Pattern*” OR “Physician Practice Pattern*” OR “Outcome Assessment” OR “Length of Stay” OR “Hospital Readmission” OR “Health Services Accessibility” OR “Health Service Accessibility” OR “Health Care Accessibility” OR “Health Services Need*” OR “Health Service Demand*” OR “Health Service Need*” OR “Health Services Demand*” OR “Health Care Need*” OR “Health Care Demand*” OR “Clinical Competence” OR “burden of disease” OR “Disease Burden” OR “model” OR “models” OR “resources” OR “resource” OR “implementation” OR implement* OR “competent” OR “productivity” OR “case mix” OR “overcrowding” OR overcrowd* OR “timing” OR “Time Factor” OR “cost” OR “costs” OR “workforce” OR “workforces” OR “trauma systems” OR “trauma system”) AND la = (english OR dutch OR german) NOT ti = ((“case report” OR “case report*”) NOT (“Review” OR “review*”)) NOT ti = (veterinary OR rabbit OR rabbits OR animal OR animals OR mouse OR mice OR rodent OR rodents OR rat OR rats OR pig OR pigs OR porcine OR horse* OR equine OR cow OR cows OR bovine OR goat OR goats OR sheep OR ovine OR canine OR dog OR dogs OR feline OR cat OR cats) AND dt = (meeting abstract)

### Cochrane

https://www.cochranelibrary.com/advanced-search/search-manager

ACS as main subject, coupled to organization and administration-terms:

(“acute care surgery” OR “acute care surgical” OR “acute care surgeons” OR “acute care surgeon” OR “acs surgery” OR “acs surgeons” OR “acute care surgery” OR “emergency surgery” OR “emergency surgical” OR “emergency surgeon” OR “emergency surgeons” OR “emergency surgeries” OR “emergency general surgery” OR “emergency general surgeon” OR “emergency general surgeons” OR “acute trauma surgery” OR “acute surgery” OR “acute surgical” OR “acute surgical care” OR “acute surgical emergencies” OR “acute surgical emergency” OR “acute surgical admission” OR “acute surgical admissions” OR “acute surgical beds” OR “acute surgical care” OR “acute surgical emergencies” OR “acute surgical emergency” OR “acute surgical intervention” OR “acute surgical interventions” OR “acute surgical management” OR “acute surgical model” OR “acute surgical patient” OR “acute surgical patients” OR “acute surgical procedure” OR “acute surgical procedures” OR “acute surgical service” OR “acute surgical services” OR “acute surgical setting” OR “acute surgical settings” OR “acute surgical site” OR “acute surgical specialties” OR “acute surgical treatment” OR “acute surgical unit” OR “acute surgical units” OR “acute surgical ward” OR “acute surgical wards” OR “surgical emergency” OR “surgical emergencies” OR “surgery emergencies” OR “surgery emergency” OR ((“Emergency Treatment” OR *”emergency care” OR *”evidence based emergency medicine” OR “Emergency Health Service”) AND (“Surgery Department” OR *”General Surgery”))):ti AND (“economics” OR “**organization and administration”** OR “standard” OR “trend study” OR “manpower” OR “Theoretical Model” OR “Educational Model” OR “nonbiological model” OR “Health Care Quality” OR “Cost–Benefit Analysis” OR “Physicians’ Practice Pattern*” OR “Physicians Practice Pattern*” OR “Physician Practice Pattern*” OR “Outcome Assessment” OR “Length of Stay” OR “Hospital Readmission” OR “Health Services Accessibility” OR “Health Service Accessibility” OR “Health Care Accessibility” OR “Health Services Need*” OR “Health Service Demand*” OR “Health Service Need*” OR “Health Services Demand*” OR “Health Care Need*” OR “Health Care Demand*” OR “Clinical Competence” OR “burden of disease” OR “Disease Burden” OR “model” OR “models” OR “resources” OR “resource” OR “implementation” OR implement* OR “competent” OR “productivity” OR “case mix” OR “overcrowding” OR overcrowd* OR “timing” OR “Time Factor” OR “cost” OR “costs” OR “workforce” OR “workforces” OR “trauma systems” OR “trauma system”):ti,ab,kw AND conference abstract:pt

## Appendix 2: Risk of bias of included studies using the Newcastle–Ottawa Scale [23]

**Table:**

References	Selection	Comparability	Outcome	Total	Quality rating
Austin et al. [5]	****	–	**	6	Low
Beardsley et al. [37]	****	–	**	6	Low
Cox et al. [38]*	–	–	–	–	
DeGirolamo et al. [30]*	–	–	–	–	–
Hsee et al. [55] (ANZ J Surg)*	–	–	–	–	–
Lancashire [43]	****		**	6	Low
Parasyn et al. [46]*	****	–	*	5	Low
Poggetti et al. [35]*	–	–	–	–	–
van Zyl et al. [33]	****	**	**	8	High
Von Conrady et al. [51]	****	–	**	6	Low
Wanis et al. [34]	****	–	**	6	Low
Britt et al. [6]	****	–	**	6	Low
Ciesla et al. [7]*	–	–	–	–	–
Dickfos et al. [39]	***	*	*	5	Low
Garland et al. [27]*	–	–	–	–	–
Hsee et al. [54] (World J Surg)	****	–	**	6	Low
Kreindler et al. [32]	****	*	**	7	Med
Lancashire et al. [43]	****	–	**	6	Low
Mathur et al. [65]*	–	–	–	–	–
Matsushima et al. [8]	****	**	**	8	High
Mpirimbanyi et al. [69]*	–	–	–	–	–
Navarro et al. [61]	****	–	**	6	Low
Poole et al. [58]*	–	–	–	–	–
Santry et al. [26]*	–	–	–	–	–
Santry et al. [25]*	–	–	–	–	–
Sorelli et al. [62]	****	–	**	6	Low
Tincknell et al. [63]*	–	–	–	–	–
Allaway et al. [36]	****	–	**	6	Low
Bokhari et al. [59]	****	–	**	6	Low
Bokhari et al. [60]	****	*	**	7	Med
Cubas et al. [14]	****	–	**	6	Low
Diaz et al. [16]	****	–	**	6	Low
Faryniuk and Hochman [31]	****	–	**	6	Low
Fu et al. [66]	****	–	**	6	Low
Gandy et al. [40]	****	–	**	6	Low
Kinnear et al. [42]	****	–	***	7	Med
Ladhani et al. [28]	****	*	**	7	Med
Lehane et al. [44]	****	–	**	6	Low
Lim et al. [9]	****	–	**	6	Low
Ekeh et al. [17]	****	–	**	6	Low
Mathur et al. [65]	****	–	**	6	Low
Musiienko et al. [45]	****	**	**	8	High
Pepingco et al. [47]	****	–	**	6	Low
Pillai et al. [56]	****	–	**	6	Low
Poh et al. [57]	****	–	**	6	Low
Qureshi et al. [15]	****	–	**	6	Low
Shakerian et al. [49] (Br J Surg)	****	**	**	8	High
Shakerian et al. [48] (World J Surg)	****	**	**	8	High
Suen et al. [53]	****	–	**	6	Low
Suhardja et al. [50]	****	–	**	6	Low
Anantha et al. [29]	****	–	**	6	Low
Barnes et al. [10]*	–	–	–	–	–
Bruns et al. [13]	***	–	**	5	Low
Miller et al. [18]	**	–	**	4	Low
Procter et al. [19]*	–	–	–	–	Low
Sweeting et al. [11]*	–	–	–	–	Low
Wang et al. [52]*	–	–	–	–	Low
Pryor et al. [20]	****	–	**	6	Low
Cherry-Bukowiec et al. [12]*	–	–	–	–	–
Guy and Lisec [41]	****	–	**	6	Low
al-Ayoubi et al. [64]*	–	–	–	–	
Dresser et al. [70]	****	–	**	6	Low
Moodie [68]*	–	–	–	–	–
Klopper et al. [67]*	–	–	–	–	–

≥8 (80%) = high; 7 (70–80%) = medium; ≤6 (<60%) = low

ANZ J Surg, ANZ Journal of Surgery; World J Surg, World Journal of Surgery

*Study designs other than case–control –or cohort studies could not be scored using the Newcastle–Ottawa Scale
